# A Scoping Review of the Operationalization of Fruit and Vegetable Variety

**DOI:** 10.3390/nu12092868

**Published:** 2020-09-19

**Authors:** Allison N. Marshall, Alexandra van den Berg, Nalini Ranjit, Deanna M. Hoelscher

**Affiliations:** Michael and Susan Dell Center for Healthy Living, University of Texas Health Science Center at Houston School of Public Health Austin, Austin, TX 78701, USA; alexandra.e.vandenberg@uth.tmc.edu (A.v.d.B.); nalini.ranjit@uth.tmc.edu (N.R.); deanna.m.hoelscher@uth.tmc.edu (D.M.H.)

**Keywords:** fruit, vegetable, variety, review, operationalization, nutrition, diversity, dietary

## Abstract

Adequate consumption of fruits and vegetables is critical for healthy growth and development. Less is known about fruit and vegetable variety, with variation in operationalization of variety. This review aims to identify currently available evidence operationalizing fruit and vegetable (FV) variety through a scoping review to summarize, compare, and critically evaluate the operationalization of variety. A secondary aim is to examine the implications of measuring FV variety and outcomes including dietary quality/nutrient intake. PubMed, Medline, PsycINFO were searched using the following criteria: (1) human study participants ages 2 years and above; (2) assessment of fruit variety consumption, vegetable variety consumption, or combined fruit and vegetable variety consumption; and (3) peer-reviewed publication available in the English language. Etiologic, intervention, and determinant studies were eligible to be included, and 47 studies met inclusion criteria. Differences in operationalization of variety were found. Findings included associations of FV variety with aspects of nutrient intake, dietary behaviors, lifestyle behaviors, and health outcomes. There were no studies that assessed conventionally grown produce vs. organic produce, and none of the included studies assessed cultivar. Nonstandard classification of fruit and vegetables, differences in fruit and vegetables grown in other countries, and the restriction to studies published in the English language may have excluded studies examining variety published in languages other than English. Operationalization of variety should be reported to allow one to explore comparability across studies, use national or international guidelines for greater comparability, associate variety with nutrient intake, and change variety behaviors via intervention.

## 1. Introduction

Adequate intake of fruit and vegetables (FV) is recognized as crucial for optimal health in childhood and adulthood and is critical for proper physical and psychosocial development, as well as functioning for children and adolescents [1]. The World Health Organization (WHO) recognizes inadequate FV consumption as a factor in 14% of gastrointestinal cancer deaths, 11% of ischemic heart disease deaths, and 9% of stroke deaths worldwide, and considers low FV intake as one of the top 10 risks for death worldwide [2]. Adequate FV intake is especially critical during childhood and adolescence because of rapid growth, and because lifestyle habits from childhood tend to track into adulthood [3]. Additionally, low FV intake is associated with higher body mass index (BMI) and higher risk of obesity in childhood [3], and childhood obesity is linked to excess weight in adolescence and adulthood [4].

FV variety affects micronutrient and macronutrient intake as different nutrients are present in different fruits and vegetables [1,5]. Increasing FV variety is positively associated with micronutrient and vitamin intake, especially beta-carotene, vitamin C, total carotenoids, vitamin A, alpha-carotene, and lutein among adults, and with higher vitamin and fiber intakes among adolescents [6].

FV, in whole, as well as through specific bioactive components they contain, provide health benefits including disease prevention; as such, they can be considered to be nutraceuticals which have been defined as functional foods that naturally have properties that promote health or prevent disease [7,8]. Nutraceuticals can range from whole foods such as FV to specific constituents, such as fiber or vitamin C. The natural bioactive compounds available in FV make consumption of FV important for health. Other aspects of FV consumption that are important to consider regarding nutrient content include distinct cultivars, breeds, varieties, or types of fruits and vegetables; organic vs. conventional growing methods and subsequent possible pesticide, herbicide, and fungicide residue; and seasonality [9,10,11].

Despite national and global efforts to increase consumption, FV intake remains below recommended levels among most adults and children [1,5,12,13]. Current recommendations for FV intake in the United States from the 2015–2020 U.S. Dietary Guidelines for Americans (USDGA) are 2.5 cup equivalents of vegetables and 2 cups of fruit per day assuming a 2000 calorie/day diet [1]. The Centers for Disease Control and Prevention estimates indicate that in 2015, 12.2% of adults met fruit recommendations and 9.3% of adults met vegetable recommendations [12]. Only 8.5% of high school students met fruit recommendations nationally, and only 2.1% met vegetable recommendations [13].

**Figure 1:**
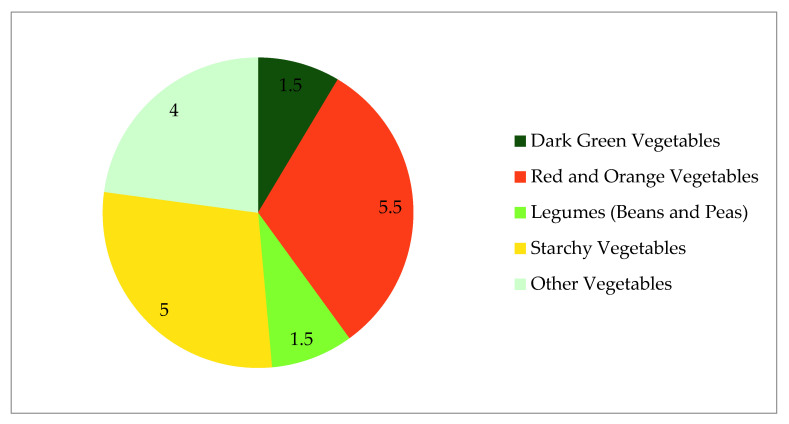
Composition of weekly vegetable variety recommendations (in cups), sum total 17.5 cups. Based on vegetable subgroup weekly recommendations for a 2000 calorie diet from the Dietary Guidelines for Americans 2015–2020.

FV recommendations from the 2015–2020 USDGA include total quantity, as well as a recommended weekly variety by vegetable subgroup [1]. Variety recommendations are available based on age and gender and are also presented based on overall caloric intake (Table 1 and Figure 1). Most age groups fail to meet variety recommendations. There is no age group of either gender that meets weekly recommendations for red and orange vegetables or dark green vegetables, and no group under the age of 30 years old meets weekly recommendations for “other” vegetables.

Although most FV recommendations encourage consumption of a variety of fruits and vegetables, there is diversity in the literature regarding what constitutes variety. This makes it difficult to compare what evidence there is regarding variety. Different researchers operationalize variety differently, creating groups and subgroups of FV based on commonly consumed foods by location, seasonality, or national guidelines [14,15,16,17,18]. The 2015–2020 USDGA groupings (Table 1) [1], have been used as the basis for some determinant studies [15,18]; however, there is no similar 2015–2020 USDGA model for subgroups of fruit intake.

Notable differences in operationalization of variety in the literature included the treatment of starchy vegetables, potatoes, legumes, and juices. Potatoes, legumes, other tubers, or vegetable juices could be included as vegetable items [1,3,6,12,13,14,15,16,17,18,19,20] or excluded [16,19,21,22]. French fries, fried potatoes, and hash browns were sometimes excluded in studies [15,23], sometimes included as a vegetable subgroup [14,24,25], or sometimes included in the starchy vegetable category [18]. Tomato and V8 juices were sometimes grouped with “other” vegetables [15], or not counted as a vegetable item [19] or considered to be a distinct subgroup [14]. Fruit juice was sometimes included within the fruit group [1,3,15,18] or other times excluded from the fruit group [19]. Citrus fruit was sometimes considered to be a distinct fruit subgroup [14,16,18] or not included at all [17]. These examples illustrate the difficulty in interpreting studies that assess FV variety.

Most studies operationalizing variety have focused on adult 2populations [14,15,16,19]. Vegetable variety was associated with total cancer and non-lung epithelial cancer; fruit variety was also associated with reduced cancer risk after excluding the first two years of followup [17]. An inverse association was found between vegetable variety with lung cancer risk among current smokers independent of the quantity consumed [16]. Provision of greater vegetable variety (multiple vegetables: broccoli, carrots, and snap peas) in an experiment significantly increased vegetable intake without increasing overall caloric intake [26].

Fewer studies have considered FV variety among children. In an experimental study, provision of greater vegetable variety (more than one) increased vegetable choice without increasing the total caloric value of the meal [27]. FV variety was associated with higher dietary quality among preschool children [18]; notably, fried potatoes were included [18] as opposed to elsewhere in the literature.

Given the differences in operationalization of variety across studies of children and adults, the aim of this paper is to identify currently available evidence operationalizing FV variety through a scoping review to summarize, compare, and critically evaluate the operationalization of variety. A secondary aim of this review is to examine and characterize the existing relations between FV variety and outcomes including nutrient intake.

## 2. Materials and Methods

**Figure 2:**
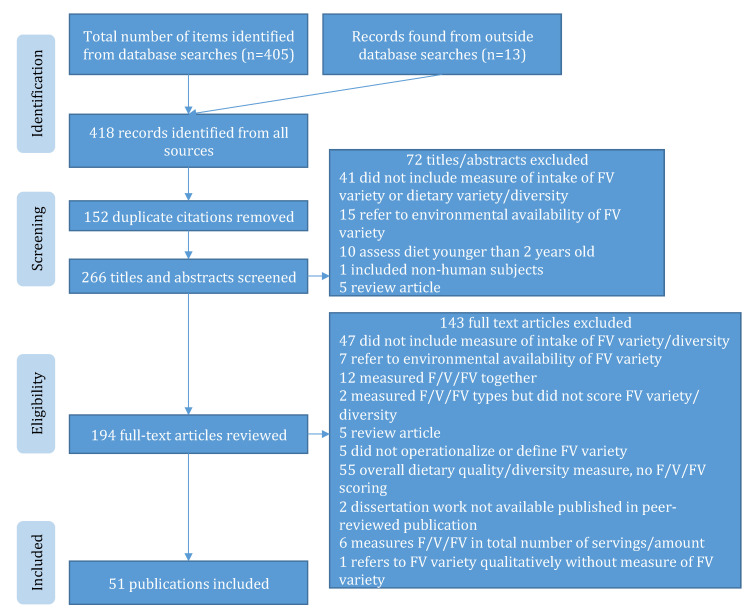
Flow diagram of methods: identification, screening, eligibility, and inclusion of records with reasons for exclusion at each stage [28,29].

This scoping review of primary, peer-reviewed literature on fruit variety, vegetable variety, and FV variety was conducted using Preferred Reporting Items for Systematic Reviews and Meta-Analyses (PRISMA) [28] and PRISMA Extension for Scoping Reviews (PRISMA-ScR) guidelines (Figure 2) [29]. The protocol for this scoping review was informed by PRISMA-P [30] and is available in Appendix A.

### 2.1. Search Strategies

Articles for this review were sourced from the following three databases: PubMed, Medline, and PsycINFO. Searches were conducted using combinations of the following terms: Fruit OR fruits OR vegetable OR vegetables AND diet OR dietary OR nutrition AND variety OR diversity AND measure OR assess. In addition, thirteen papers from the authors’ existing literature base and citations from screened papers were included in screening.

### 2.2. Inclusion and Exclusion Criteria

All studies were evaluated using the following inclusion criteria: (1) human study participants ages two years old and above; (2) assessment of at least one of fruit variety consumption, vegetable variety consumption, or combined FV variety consumption; and (3) peer-reviewed publication available in the English language. Etiologic, intervention, and determinant studies were eligible to be included.

### 2.3. Study Selection

Studies were screened, duplicates were removed, and titles and abstracts were scanned for relevancy to identify a list of potentially relevant studies. Articles not meeting the inclusion criteria were removed and reasons for exclusion were noted. The scoping review and selection processes of the study are shown in the flow diagram (Figure 2) [28,29].

### 2.4. Data Extraction Techniques

Key information was collected from included studies using a data extraction table. Author, publication year, study design, sample size, sample characteristics, operationalization of variety, number of FV groups or items and specific items when reported, and findings from each study were included in the data extraction table.

## 3. Results

Initial searches yielded a total of 405 papers from PubMed (*n* = 184), Medline (*n* = 178), and PsycINFO (*n* = 43); an additional thirteen manuscripts were identified from previous research or reference lists of other manuscripts; and a total of 418 records were identified from all sources. Removal of duplicates (*n* = 152) left 266 unique papers to be screened. Screening abstracts resulted in exclusion of an additional 72 items; a total of 194 papers went to full-text review. A total of 51 studies met all inclusion criteria for this review, presented in Table 2. The most common reason for exclusion during both abstract and full-text screening was a lack of measurement of fruit, vegetables, or FV variety intake. Additionally, common during the abstract screening was a focus on fruit, vegetables, or FV variety at the environmental level in terms of availability without assessment of individual dietary intake (*n* = 15); an additional seven studies were excluded during the full-text screening stage for this reason.

During full-text screening, another common reason for exclusion was a measure of overall dietary variety or quality without specific fruit, vegetables, or FV variety or within-group diversity. Many papers (*n* = 55) included an assessment of dietary diversity or food variety scores that considered the number of different food items eaten or the number of food groups represented in a person’s diet over a period of time, but do not report scores for variety within F, V, or a combined FV group.

### 3.1. Study Design and Samples

Sample size varied widely as shown in Table 2, ranging from the largest sample size of *n* = 521,448 in a 10-year prospective cohort study [31], to the smallest sample size of *n* = 18 [32]. Participant age varied widely; the youngest participants were 2 years old [18], and the oldest participants were 84 years old [17]. Three studies included child participants [18,33,34] and four studies included adolescent participants [35,36,37,38]. Thirty-seven studies included adult participants [14,15,16,17,19,21,22,23,24,25,26,31,32,39,40,41,42,43,44,45,46,47,48,49,50,51,52,53,54,55,56,57,58,59,60,61,62]; seven studies included a combination of children or adolescents and adults [63,64,65,66,67,68,69].

Studies from across the world were included in this review, including Europe (France, Germany, Greece, Italy, Netherlands, Portugal, Spain, and the United Kingdom), North America (Canada, United States, Puerto Rico), Central America (Guatemala), South America (Brazil), Australia, Africa (Ghana, Mali), and Asia (Iran, Korea).

Study design was not explicitly reported for a few of the included studies (*n* = 2), one of which indicated that the study design was reported elsewhere. All study designs were eligible for inclusion in this review.

The cross-sectional study design was the most common design among the included studies (Table 3), reported in 23 papers [15,18,34,35,36,38,39,40,41,42,43,44,45,47,51,52,53,58,63,64,65,67,68]. These studies included analysis of existing, ongoing national surveillance datasets in the United States [15,18,44], cross-sectional studies in USA [47,51], and in international populations [34,35,36,38,39,40,41,42,43,45,52,63,65,67]. Cross-sectional analyses from prospective cohort studies were also included [40,43].

Eight distinct prospective cohort studies were represented by ten included papers (Table 4) [16,17,19,21,22,31,48,59,61,62]. One paper presented the following two prospective cohorts: a nurses’ health study (*n* = 71,141 women), and the health professionals’ follow-up study (*n* = 42,135 men) [61]. One paper presented analyses of subjects within the Korean Hereditary Breast Cancer (KOHBRA) Study, a prospective cohort study (*n* = 2271) examining the association between dietary behaviors and breast cancer risk [48]. One paper presented results from a French prospective cohort study (*n* = 4282), in which socioeconomic, demographic, and behavioral factors were examined in relation to FV variety and quantity consumption [22]. One prospective cohort study was conducted with community-dwelling elders in Taipei to examine the association of diet quality and V variety with cognitive decline [62]. Two Dutch prospective cohort studies were presented, part of the Zutphen study (*n* = 730 Dutch men) in which FV quantity and variety were examined in relation to cancer, [17] and the Monitoring Project on Risk Factors and Chronic Diseases in the Netherlands (MORGEN) Study (*n* = 20,069), in which the importance of FV variety was examined relative to the incidence of coronary heart disease (CHD) and stroke [19]. Four of the included cohort papers presented results from the same larger prospective cohort study of 10 European countries, the European Prospective Investigation into Cancer and Nutrition (EPIC) [16,21,31,59]. Among the studies that used EPIC data, FV variety consumption was examined as related to lung cancer (*n* = 452,187) [16], bladder cancer (*n* = 452,185) [59], and colon and rectal cancer (*n* = 521,488) [31]. The third study that used EPIC data was a prospective case cohort from the Norfolk component of EPIC, in which the 11 year incidence of type 2 diabetes was examined in relation to the amount and variety of fruit, vegetables, and FV consumed among 3704 participants, 653 of which were diabetes cases [21]. Cohort studies are presented in Table 4.

The remaining observational papers used case-control and case-only study designs (Table 5) [46,50,55]. One paper included a study sample of men and women with colon cancer (*n* = 428) [50]. An additional paper presented results from analyses of colon cancer controls from within a case-control study [55]. One paper with a case-only design included women with breast cancer [46].

Ten of the included papers were experimental, and represented distinct studies [14,23,26,32,33,49,54,56,60,66]; four utilized a randomized controlled design [14,23,49,66]. All findings presented in the included papers indicated increased FV variety among participants receiving interventions [14,23,33,49,66]. Randomized controlled trials (RCTs) were used to test the following: a multistate tailored intervention with low-income adults (*n* = 1255) [14]; an intervention to increase vegetable intake, liking, and variety among families with a 9–12-year-old child using food assistance (*n* = 46 families, 36 intervention, 10 control) [66]; a computer-tailored newsletter intervention to increase FV variety among adults (*n* = 573) [49]; and to compare two different dietary approaches (*n* = 120) [23]. Additionally, a crossover control design (*n* = 18) was used to test an intervention to increase FV consumption and improve dietary quality and it was found that patients who received the intervention increased their intake of fruit variety [32]. Counter-balanced crossover design was used in two studies [26,56]; authors found greater fruit variety increased fruit consumption in the last course of fruit offered (*n* = 20), [56] and increased vegetable variety intake increased vegetable variety served at meals with (*n* = 66) [26]. Another crossover study found that an increased variety of F (three types at once) and increased variety of V (three types at once) increased selection of FV variety by children, and increased consumption [33].

### 3.2. Measurement Instruments

The most commonly used measurement instruments among included studies were FFQs (*n* = 24) [14,16,17,19,24,35,37,40,43,46,47,48,49,51,55,57,58,59,61,62,63,64,65,68] and 24-h dietary recalls (*n* = 13) [18,22,23,32,34,38,39,44,52,53,60,66,68], in addition to brief FV questionnaire instruments (*n* = 4), food records (*n* = 3) or food diaries (*n* = 1), weighted food records (*n* = 1), interviews (*n* = 3), and variations of food item checklists. Some experimental studies used food weights to measure consumption (*n* = 3) or frequency counts (*n* = 1). The level of detail provided on measurement instruments and administration varied from very little to a thorough description of the measurement instrument including title, number of items, method of administration, and structure of questions, and response options. Additionally, studies often included more than one type of measurement instrument [23,31,50,57,66].

#### 3.2.1. Food Frequency Questionnaires (FFQs)

Twenty-five of the included studies used some variation of a Food Frequency Questionnaire [14,16,17,19,24,31,35,37,40,43,46,47,48,49,51,55,57,58,59,62,63,64,65,68], including quantitative [68], semiquantitative [43,58,61,62], self-administered, and interviewer administered [14,50,58]. A combination of different instruments and administration methods were used in a 10-country prospective cohort study, which varied by country [16,31,59]. Interviewer administration of FFQs was conducted in-person, and over the phone. Some authors did not report the specific type of FFQ or mode of administration or the number of items on the FFQ used, however, those that did indicate varied lengths of FFQs, the shortest of which was 17 FV items. Notably, the longest reported FFQ of 223 items was interviewer administered in-home to adults 45–75 years old [58]. FFQs were most often used with samples of adults but were also used with adolescents and children.

#### 3.2.2. Twenty-Four-Hour Dietary Recalls

Among the thirteen studies using 24-h dietary recalls as measurement instruments, there was variation in the number of records included in each study, administration methods, and the treatment of a single 24-h recall [18,22,23,32,34,38,39,44,52,53,60,66,68]. Recalls were self-administered and verified by the nutritionist [34], interviewer administered [18,23,32,38,39,44,52,53,60,69], and conducted through telephone/software systems [22]. Four papers utilized a single 24-h dietary recall [18,34,44,53]. Three of the studies used two 24-h dietary recalls to assess usual diet [39,52,69], which were averaged [69] or assessed individually [23,60].

Two studies used three 24-h dietary recalls [32,38], one study used four [23], and another used a series of six recalls over a period of two years [22]. The most 24-h dietary recalls used in a single study was 8–12 over four months, with a variable number of recalls per participant [60]. With regards to study sample, 24-h dietary recalls were used with children in three studies [18,34,66], adolescents in two studies [38,69], and adults in nine studies [22,23,32,38,39,44,52,60,69].

#### 3.2.3. Other Measurements

Less commonly used measurement instruments included a Dietary Variety Questionnaire which included a checklist of FV over three days [41,42], a Brief Evaluation Questionnaire on Fruit and Vegetable Consumption (QBrief-FV) [45], FV checklists [25,32], and seven-day food diaries [21]. Studies conducted with Australian participants included the Australian Recommended Food Score [36,67]. Within a large prospective cohort study, food records and interviews were used for some groups [16,59]. 

Five authors reported the use of multiple instruments within participants [23,31,50,57,66]. In two cases, three 24-h recalls were combined with other instruments, one case with the use of a FV checklist [32], and the other case a measurement instrument adapted using 36 vegetables from an existing questionnaire (more details not reported) [66]. In a large prospective cohort study, dietary questionnaires were combined with 7-day food records for two groups within the cohort [31]. One other study included calculation of the Healthy Eating Index for Australian Adults, a weighed food record (WFR), and FFQ [57]. In experimental studies to measure actual consumption, food items were weighed before and after consumption [26,33,54,56].

### 3.3. Quantifying Variety

#### 3.3.1. Timeframe and Frequency

The timeframe measured in the studies varied from 24-h dietary recalls to usual intake referring to the previous year. Fourteen of the studies referred to usual intake over the previous year [14,16,19,35,40,43,46,48,50,55,58,59,63,64]. Of studies referring to usual intake over the previous year, two studies required a minimum frequency of at least once per week in the previous year [46,48], four required at least once per two weeks in the previous year [16,19,31,40,59] (four of which used data from the same prospective cohort study) [16,40,59], three required at least once per month in the previous year [14,35,58], and one required consumption more than once per month [55]; others did not report a frequency required other than referring to usual intake over the previous year [43,50,63,64]. Chou et al. (2019) scored V variety based on cup-equivalents per week [62]. Another study did not report the timeframe assessed by the semiquantitative FFQ, but required consumption at least once per week to be included in variety, which was, then, used to calculate average daily consumption [61].

Usual intake was also considered over a period of six months [45,51], four months [60], three months [24,37], four weeks [26,47], past month [25,57,65,66], fifteen days [32], a week [21,49,68], past three days [41,42], a two-day period [52], and a single 24-h period [18,34,38,44,53].

#### 3.3.2. Minimum Amount

There was also variation in the importance of a minimum amount required to be considered for variety. Many authors did not report a minimum amount of fruit, vegetables, or FV required to be included in a measurement of variety [16,17,18,19,22,23,24,32,37,38,40,44,46,47,48,49,51,55,59,60,66,68,69].

Other authors indicated that any amount of a fruit or vegetables item is sufficient to be counted as a unique FV item which would constitute variety and that no minimum amount of any given FV item was necessary [21,25,41,42,43,65]. Experimental studies which weighed foods before and after eating considered any amount of consumption as consuming variety [26,33,54,56].

Among authors that considered a minimum amount to count towards variety, seven made reference to serving sizes, in whole or as a proportion, setting a minimum of a half serving size, [39,52,53,63,64,65] or a full standard serving size [14,57]. Some authors specified different serving sizes for fruit and vegetables as follows: at least 50% of a serving, or ≥75 g of fruit and ≥37.5 g of vegetable [53]. Another author required a minimum of one serving size of vegetable (75 g) or a half serving size of legumes and one serving of fruit (150 g) [57]. National and international recommendations [34] were commonly cited as the basis for serving size, including the U.S. Department of Agriculture (USDA) Food Guide Pyramid [62,63,64] and MyPlate [18].

### 3.4. Fruit and Vegetable Items and Subgroups

FV subgroups were frequently based on national guidelines, reflecting changes in guidelines over time across studies. Several study authors did not report the number of fruit items, vegetable items, or FV combined items [32,36,38,44,46,49,55,57,60,67,68]. The level of detail reported on specific FV items measured varied greatly. The fewest number of fruit items measured was two [15,39,52,63,64], and the maximum number of fruit items was 58 [21]. Maximum numbers of vegetable items measured ranged from five [41,42] to 59 [21]. Some studies only considered fruit and vegetables separately, whereas others considered FV combined (*n* = 12), either solely (*n* = 5), or in addition to fruit and vegetables separately (*n* = 7). Reporting of FV combined items ranged from a maximum of 10–117 items.

#### 2015–2020 U.S. Dietary Guidelines for Americans: Vegetable Subgroups

Within this scoping review, no studies used the exact five vegetable subgroups consistent with the USDA dark leafy greens, red and orange vegetables, legumes, starchy vegetables, and other vegetables [1,3]. However, there were similar groupings. Conrad et al. (2018) and Ramsay et al. (2017) used closely aligned subgroups, with the exception of legumes, which were not included, and the inclusion of white potatoes and French fries with the starchy vegetable group [18,44]. Roy et al. (2016) classified vegetables as green, orange, cruciferous, tubers, and bulb and legumes [57]. Brunt et al. (2008) classified vegetable as green leafy vegetables, orange and yellow vegetables, tomatoes and tomato products, potatoes and other root crops, and other vegetables [41,42]. Sidahmed et al. (2014) also used similar vegetable groups, including deep green, deep yellow, tomato, white potato, other starchy vegetables, other vegetables, fried vegetables not including potatoes, and vegetable juice, thus, separating white potatoes into a separate vegetable group, including the addition of fried vegetables as a group, and of vegetable juice as a separate group [23]. Chou et al. (2019) noted the USDGA subgroups as foundation for the study, but used the following five vegetable subgroups: spinach and broccoli; other dark-green vegetables; red and orange vegetables; starchy vegetables; and other vegetables [62]. 

### 3.5. Seasonality, Dietary Differences by Country and Region

Part of the challenge in operationalizing FV variety was that FV variety varied by country, region, and season. Seasonality was considered in several of the included articles. Three studies tailored the FV items to the specific countries in which the studies were conducted, as well as seasonality [17,19,22]; assessment was conducted over different seasons to capture changes in season [19,22]. Another study conducted subsequent analyses to examine seasonality [14]. Others considered seasonality in an aspect of variable creation/measurement [21,35]. Additionally, in the EPIC study, which included 10 countries, FV measurement instruments and items included varied by country; analyses were conducted using items which were common among the countries [16,31,59]. Because thirteen of the studies referred to usual intake over the previous year [14,16,35,40,43,46,48,50,55,58,59,63,64], all seasons should be included.

### 3.6. FV Variety and Outcomes

Variety and nutrient intake were correlated in several studies [39,52,55,69]. Fruit variety was correlated with vitamin C intake, and with the probability of vitamin A, vitamin C, and potassium adequacy (*n* = 295 men) [39]. Mirmiran et al. (2006) found that, among a sample of 286 adult women, fruit diversity was correlated with vitamin C intake, and the probability of vitamin A, vitamin C, and potassium adequacy [52]. Vegetable variety correlated with vitamin A, potassium, and vitamin C adequacy in multiple studies (*n* = 295 men aged 18 and older) [39] and (*n* = 286 women) [52]. Randall et al. (1989) found total diversity, fruit diversity, and vegetable diversity scores were associated with fiber, vitamin A, and vitamin C intake (*n* = 428) [55]. Oude Griep et al. (2012) found FV variety was positively associated with vitamin C, flavonoids, and dietary fiber intake [19]. Notably, none of the included studies assessed the growing methods, i.e., organically or conventionally produced or any pesticide, herbicide, or fungicide residue; also none of the included studies assessed cultivars, breeds, types, or species of FV. In addition, most studies considered FV by groupings and assigned equal value with regards to diversity in terms of frozen, raw, or canned FV.

FV variety was linked to multiple aspects of healthy dietary behaviors in several studies. Vandevijvere et al. (2010) found vegetable diversity was positively associated with meeting recommended intake for the vegetable food group among a Belgian sample (*n* = 3245) [69]. Torheim et al. (2004) found a high correlation between mean adequacy ratio (MAR) and vegetable variety among 503 women in Mali [68]. Azadbakht et al. (2013) found that among Isfahanian women aged 12–28 years old (*n* = 411), those who consumed breakfast had higher diversity scores for both fruit and vegetables [63]. In a similar sample, Azadbakht et al. (2012) found that women in the top tertile for overall dietary diversity scores had the highest fruit diversity and vegetable diversity scores [64]. Oude Griep et al. (2012) found that FV variety was strongly correlated with total FV intake (Spearman’s r = 0.81, *p* < 0.0001) [19]. Similarly, Bhupathiraju et al. (2013) found that a higher FV intake was associated with higher FV variety, and participants with higher quantity-adjusted variety scores had higher FV intakes [61]. Keim et al. (2013) found that women with higher vegetable variety intake had better indices of diet quality, more healthy attitudes about food and eating, and allocated more money towards food including vegetables [24]. Leenders et al. (2015) found higher FV variety was associated with higher absolute consumption of FV [31].

FV variety was found to be correlated with lifestyle habits across studies. Estaquio et al. (2008) found fruit variety was associated with nonsmoking in men and women, as well as with regular physical activity and low alcohol consumption in men; vegetable variety had an inverse relationship with smoking among men (*n* = 4282) [22]. Among 38,981 U.S. adults, Conrad et al. (2018) found current smoking was associated with lower vegetable variety [44]. Buchner et al. (2011) found that individuals with higher FV variety were more often never smokers and had lower BMIs (*n* = 452,185) [59]. Likewise, Oude Griep et al. (2012) found that those consuming a greater variety of FV were more likely to be physically active and to be non-smokers [19]. Bhupathiraju et al. (2013) found that higher quantity-adjusted variety scores were associated with lower likelihood of smoking, more physical activity, and higher FV intake, among other healthy lifestyle behaviors [61].

Several studies described significant associations among FV varieties and sociodemographic factors. Among 4282 French adults, Estaquio et al. (2008) found significant positive relationships between vegetable variety and education among both men and women [22]; fruit variety was positively associated with education and occupation in men [22]. Low education and low social class were associated with less fruit variety and less vegetable variety among 9850 English adults over aged 50 [43]. In a study of 654 Australian adolescents and 7695 Australian adults, Giskes et al. (2002) found lower FV variety consumption among lower-income adults as compared with higher-income adults, but this relationship was only significant among adults [38].

Bonaccio et al. (2018) found that FV variety was positively associated with psychological resilience among Italian men and women aged 35 years and older (*n* = 10,812) [40]. Azupogo et al. (2018) found increasing trends across vegetable variety score tertiles for health-related quality-of-life (HRQoL) (*p* trend = 0.0003), physical health (*p* trend = 0.02), mental health (*p* trend = 0.001), and physical functioning (*p* trend = 0.01) among women in rural Ghana (*n* = 187) [65]. Among community-dwelling older adults, Chou et al. (2019) found no significant associations between quantity-adjusted vegetable variety with risk of cognitive decline [62]; however, high diet quality was associated with lower risk of global cognitive decline among elders consuming high vegetable variety [62]. 

Findings regarding cancer were somewhat mixed and varied by type of cancer. In a case-only study of 739 women, Ghadirian et al. (2009) found a strong significant interaction between BRCA mutations and FV diversity between upper and lower quartiles [46]. Vegetable variety was inversely associated with lung cancer risk among current smokers [16]. Buchner et al. (2011) did not find any clear association between FV variety consumption and bladder cancer risk; the highest tertile of scores for FV consumption had a marginally significant hazard ratio as compared with the lowest (HR = 1.30, 95% CI: 1.00–1.69, *p*-trend = 0.05) (*n* = 452185) (2011) [59]. Oude Griep et al. (2012) found no significant associations among fruit variety, vegetable variety, or FV variety with either incident of coronary heart disease (CHD) or stroke after adjusting for dietary and lifestyle factors [19]. Likewise, Bhupathiraju et al. (2013) found no associations among quantity adjusted FV variety score with risk of CHD, or with nonfatal myocardial infarction, or fatal CHD [61]. Leenders et al. (2015) found no association found between FV variety and risk of developing colon cancer, and increased risk of rectal cancer with higher F variety [31]. However, Leenders et al. also found higher self-reported FV consumption associated with lower risk of colon cancer (HR Q4 vs Q1 0.87, 95%C! 0.75-2.02, *p* for trend 0.02) [31].

Cooper et al. (2012) found associations of greater fruit variety (0.70 [0.53–0.91]), greater vegetable variety (0.77 [0.61–0.98]), and combined FV (0.61 [0.48–0.78]) with lower hazard ratios of type 2 diabetes [21]. Haws et al. (2017) found that vegetable variety was a significant mediator of weight loss over time among women with overweight/obesity (β = −0.357, *t*(946) = 3.02, *p* = 0.003) (*n* = 134) [60].

Fruit variety, vegetable variety, and FV variety can be increased by interventions. Do et al. (2008) utilized an RCT design with *n* = 1255 low-income adults aged 18–24 and found significantly greater fruit variety and vegetable variety among participants receiving the intervention [14]. Lutz et al. also found higher FV intake and variety among participants in the experimental condition in an RCT (*n* = 573) [49]. In a small (*n* = 18) crossover-controlled design. Falciglia et al. (2005) found increased fruit variety intake among participants that received an intervention to increase FV consumption and improve dietary quality [32]. Additionally, increasing environmental FV variety could be a useful strategy to increase FV variety consumption; in a study examining FV variety consumption, purchasing characteristics, and food environment, de Deus Mendonça et al. (2019) posited that greater fruit variety in commercial outlets would increase fruit variety consumption [45].

## 4. Discussion

Within this scoping review as compared with studies examining FV intake, relatively few FV studies examined the role of variety. Notably, virtually all of the studies had differing definitions for FV variety, making it difficult to compare study results and to make general recommendations. In addition, most studies were conducted among adults, with few assessing associations of FV variety in children and adolescents. Although studies showed a significant positive correlation between FV variety and FV intake, there were differences in associations among FV variety and health outcomes as compared to those found among FV intake and health outcomes. FV variety should be examined within the continuum of nutraceuticals, which ranges from whole foods to their constituents. This study focused only on whole FV consumption since the intent was to capture a broad overview of the operationalization of FV variety in the literature. Future studies should consider a more inclusive range of nutraceuticals in terms of composition and components. More detailed analysis of the composition of FV variety could help to elucidate which nutraceuticals are linked to specific health outcomes.

An assessment of FV variety based on national or international guidelines would allow researchers to make comparisons across studies. Seasonality and country- and region-specific data should be collected when possible for context about FV variety and for improved comparability and generalizability of findings. Additionally, measurement and reporting of specific items of FV could allow for comparability across different guidelines, studies, and different countries, and allow for consideration of differences in FV variety by country, region, and season. Assessment of cultivars would provide greater context for understanding FV consumption and allow for greater understanding of the importance of cultivars and associated nutrient content [10,11], which could influence the significance of findings of associations among FV consumption and health outcomes including nutrient intake. Another aspect of FV consumption which must be considered is consumption relative to seasonality, i.e., when FV are harvested and transported, which can lead to nutrient degradation over time [9]. Consumption of local, seasonal FV is one way to minimize nutrient degradation. Additionally, further studies should examine the differences in nutrient content comparing fresh, frozen, and canned FV, as few studies have done so [9].

There is a need for further examination of FV variety. FV variety should be assessed across the lifespan, including behaviors and health outcomes. Increased understanding of FV variety in childhood and adolescence is especially critical, considering the effects of childhood health behaviors into adulthood. Furthermore, although FV variety and FV intake have been found to be correlated, the differences in findings regarding FV variety and FV intake warrant further exploration of FV variety in addition to intake in terms of quantity.

### Strengths and Limitations

A strength of this review is the use of the standardized PRISMA guidelines [28], the PRISMA Extension for Scoping Reviews (PRISMA-ScR) guidelines [29] as well as the protocol development informed by PRISMA-P [30].There have been no published literature reviews of FV variety, so this review fills a gap in the literature. A limitation of the study is a possibility of missing some relevant studies due to nonstandard classifications and nonstandard identification of the construct of FV variety, which persists even after careful screening of abstracts and full-text papers. Additionally, this review is restricted to studies published in the English language, which excludes studies examining variety in other countries that are published in languages other than English.

Most included studies relied on FFQs and dietary recalls, which could be prone to recall bias due to the retrospective nature of measurement, and FFQs were prone to bias due to long periods of time to which they often refer [70]. Furthermore, due to the self-report nature of these methods of dietary assessment, there was potential for social desirability bias [71]. Several studies utilized a combination of multiple assessment methods [23,31,50,57,66], which was considered to strengthen the measurement within a single study [70]. FFQs are often used because of the reduced burden on participants, and the fact that they are cost-effective and time saving, and they have established utility [70,72]. Some of the included studies used interviewer administered FFQs and dietary recalls, which could improve accuracy and minimize recall bias [70,72]. Another measurement concern is the grouping of FV items together, which could result in lower variety scores, and omission of some FV items or groups could also result in lower variety scores [49].

None of the included studies reported assessment of the use of conventional or organic produce. This could be a limitation with regards to reporting the findings of individual studies, as pesticide residue and other aspects of growing and harvesting conditions could influence nutrient content or intake [73]. Additionally, none of the included studies reported measurement of cultivars, or even breed or species of FV, which could affect nutrient contents of FV [10]. However, few self-report instruments measured specifics of FV beyond type, and the included studies were intended to assess overall dietary intake of FV. Challenges in measuring cultivars include that respondents are not always able to identify a specific variety, cultivar, or breed of an item; and that specific expertise may be required to confirm correct identification of cultivars, and names for cultivars or varieties can vary by location and culture [11]. The FAO and Biodiversity International acknowledge both the challenges of measuring cultivars and the importance of better understanding of food composition including biodiversity at the level of cultivar or variety and provide guidelines for measurement of cultivars and biodiversity in dietary intake [11]. More detailed assessment of cultivars or varieties of FV should be considered for future research on dietary intake, especially as related to FV variety intake.

FV variety should be examined within the continuum of nutraceuticals, which ranges from whole foods to their constituents. This study focused only on whole FV consumption, since the intent was to capture a broad overview of the operationalization of FV variety in the literature. Future studies should consider a more inclusive range of nutraceuticals in terms of composition and components. More detailed analysis of the composition of FV variety could help to elucidate which nutraceuticals are linked to specific health outcomes.

Another problem with FV variety, in general, is that the variety of fruit, vegetables, or combined FV can vary by country and/or season, and therefore it can be difficult to compare across countries. However, several of the included studies have considered this by using country-specific measures [16,17,59] and accounting for seasonality timing of measurement or in subsequent analyses [14,17,22,51]. This should be considered in future research.

## 5. Conclusions

There is substantial variation in the operationalization of fruit, vegetables, and FV variety. However, some commonalities can be found in the use of instruments, for example, FFQs and 24-h dietary recalls are often used to measure FV variety in the included studies. Overall, most study authors reported sufficient detail on the FV items assessed, to allow us to conduct some degree of comparison across studies. This literature review helps to elucidate the current uses of FV variety as operationalized in the literature, as well as to compare results across studies. Detailed and standardized reporting of FV items and groupings is needed; more detailed measurement including assessment of cultivars is also needed. Although there were some commonalities between the groupings used in different papers, most were due to using the same or similar datasets. A more consistent operationalization of FV variety would allow us to conduct a better comparison across studies to further the understanding of the role and importance of FV variety in health promotion assessment and interventions.

## Figures and Tables

**Table 1:** Vegetable subgroup weekly recommendations for a 2000 calorie diet and examples from the Dietary Guidelines for Americans 2015–2020.

Vegetable Subgroup	Weekly Intake	Example Items (Not Exhaustive)
Dark green vegetables	1.5 cups	Broccoli, spinach, leafy salad greens (including romaine lettuce), collards, bok choy, kale, turnip greens, mustard greens, green herbs (parsley and cilantro)
Red and orange vegetables	5.5 cups	Tomatoes, carrots, tomato juice, sweet potatoes, red peppers (hot and sweet), winter squash, pumpkin
Legumes (beans and peas)	1.5 cups	Pinto, white, kidney, and black beans; lentils; chickpeas; limas (mature and dried); split peas; edamame (green soybeans)
Starchy vegetables	5 cups	Potatoes, corn, green peas, limas (green and immature), plaintains, cassava
Other vegetables	4 cups	Lettuce (iceberg), onions, green beans, cucumbers, celery, green peppers, cabbage, mushrooms, avocado, summer squash (includes zucchini), cauliflower, eggplant, garlic, bean sprouts, olives, asparagus, peapods (snowpeas), beets

**Table 2:** Overview of studies meeting the inclusion criteria.

Citation	Sample	Measurement Instrument Type	Timeframe	Number of F, V, FV Items/Groups
Almeida-de-Souza et al., 2018	*n* = 412 adolescents ages 14.4 ± 1.7 years; 52% girls; Portugal	FFQ	1 year	12 F; 15 V
Azadbakht et al., 2013	*n* = 411 Isfahanian women aged 12–28 years	FFQ	1 year	2 F; 7 V
Azadbakht et al., 2012	*n* = 289 Isfahanian women aged 12–28 years	FFQ	1 year	2 F; 7 V
Azadbakht et al., 2005	*n* = 295 males ≥ 18 years old, (20% 51 or older); Tehran, Iran	24-h recall	2 days	2 F; 7 V
Azupogo et al., 2018	*n* = 187 women age 15–49 in rural Ghana	FFQ	1 mo	27 V
Bhupathiraju et al., 2013	*n* = 113,276 adults, U.S.	FFQ	NR	11 F; 19 V
Bonaccio et al., 2018	*n* = 10,812 men and women ≥ 35 years old, Italy	FFQ	1 year	37 FV
Brunt et al., 2008	*n* = 557 Canadian college students 18–56 years old, 60% female	DVQ	3 days	5 F; 5 V
Brunt et al., 2008	*n* = 585 college students 18–56 years old	Q	3 days	5 F; 5 V; 10 FV total
Buchner et al., 2010	*n* = 452,187 participants from 10 European countries	varied, FFQs, food records, interviews	1 year	14 F; 8 V; FV (NR); 26 V products
Buchner et al., 2011	*n* = 452,185 participants from 10 European countries	varied, FFQs, food records, interviews	1 year	14 F; 8 V; FV (NR); 26 V products
Burrows et al., 2016	*n* = 25 competitive adolescent male 14–18-year-old rugby players	ARFS	1 week	F(NR); V(NR)
Chou et al., 2019	*n* = 436 elders in Taipei	FFQ	1 year	F(NR); V(NR); 5 V subgroups
Conklin et al., 2015	*n* = 9580 over-50 s in EPIC-Norfolk (England)	FFQ	1 year	11 F; 26 V
Conrad et al., 2018	*n* = 38,981 adults > 20 years old	24 h recall	1 day	V(NR)
Cooper et al., 2012	*n* = 3704; 653 diabetes nested cases: EPIC and Nutrition-Norfolk; Norfolk, England	7 day food diaries	7 days	58 F; 59 V; 117 FV total
de Deus Mendonça et al., 2019	*n* = 3414 adults, 88.1% women ≥ 20 years old; Brazil	Q	6 mos	14 F; 22 V; FV (NR)
Do et al., 2008	*n* = 1255 low-income adults aged 18–24 years	FFQ	1 year	12 F; 14 V
Estaquio et al., 2008	*n* = 4282 French men and women 45–62 years old	24-h recall	6 days	9 F; 10 V
Falciglia et al., 2005	*n* = 18, 33–79 years old; 100% white	24-h recall	15 days	F, V, FV (NR)
Galloway et al., 2003	*n* = 192 7-year old girls and their parents; Pennsylvania, US	FFQ	3 mos	20 V
Ghadirian et al., 2009	*n* = 739 women in original cohort; mean age 50.5 for BRCA carriers, 53.4 for non-carriers	FFQ	1 year	VF (NR)
Giskes et al., 2002	*n* = 654 13–17 years old; *n* = 7695 18–64 years old; Australia	24-h recall	1 day	F(NR); V(NR)
Haws et al., 2017	*n* = 134 women with overweight/obesity	24-h recall	4 mos	V (NR);
Henry et al., 2006	*n* = 420 low-income, African American mothers 18–45 years old with children < 12 years old; United States	FFQ	4 wks	20 F; 23 V
Jansen et al., 2004	*n* = 730 Dutch men 65–84 years old followed for 10 years	FFQ	1 mo	7 F; 27 V
Keim et al., 2014	*n* = 112 low-income women 20–55 years old, BMI 11.7–68.5, primary food purchasers/preparers, California	FFQ	3 mos	21 V
Ko et al., 2013	*n* = 2271 subjects from (KOHBRA) Study; at study entry, mean age of 42.5 years old for BRCA carriers, 41.9 for non-carriers	FFQ	1 year	12 F; 25 V
Leak et al., 2015	*n* = 46, families with 9–12-year-old children (36 intervention, 10 control)	Q; 24-h recall	30 days	36 V
Leenders et al., 2015	*n* = 521,448 participants from 10 European countries	varied, FFQs, food records, interviews	1 year	FV DDS: 49 items F DDS: 16 items V DDS: 33 items V subtype DDS: 8 subtypes
Lutz et al., 1999	*n* = 710 at baseline; *n* = 573 post-intervention survey; adults	FFQ	1 wk	FV (NR)
McCann et al., 1994	*n* = 428 (205 men, 223 women) all Caucasian; Western New York, US	Interview, FF instrument	1 year	38 F; 20 V
McCrory et al., 1999	*n* = 71 men and women 20–80 years old; New England, US	FFQ	6 mos	10 F; 14 V
Meengs et al., 2012	*n* = 66 (32 men aged 20–45; 34 women aged 20–45) Pennsylvania, US	Food weights	4 wks	3 V
Mirmiran et al., 2006	*n* = 286 Tehranian women 18–80 years old	24-h recall	2 days	2 F; 7 V
Nour et al., 2017	*n* = 2397 ages 18–34 years in rural and metropolitan Australia	24-h recall	1 day	6 F; 6 V
Oude Griep et al., 2012	*n* = 20,069 (8988 men, 11081 women); Dutch	FFQ	1 year	9 F; 13 V; 22 FV total
Parizel et al., 2017	*n* = 59 healthy weight French adults 18–40 years old	freq. count	4 sessions	1–3 V
Ramsay et al., 2017	*n* = 2595 ages 2–5 years; 48% male; 55% non-Hispanic white, US.	24-h recall	1 day	4 V, 3 F
Randall et al., 1989	*n* = 428 (205 men, 223 women)	FFQ	1 year	F (NR); V (NR)
Raynor et al., 2012	*n* = 20, 50% female, 100% non-Hispanic white, mean age 26.5 years; Rhode Island, US	Food weights	four 7-min courses	4 F
Robinson et al., 2015	*n* = 66 families with parent-child dyads 8–12 years old	70-item ARFS	NR	F(NR); V(NR)
Roe et al., 2013	*n* = 61 children, age 3–5 years	Food weights	8 sessions	3 F, 3 V
Roy et al., 2016	*n* = 100 young adults from university student population, representative sample; mean age 23.5, range 18–34 years	FFQ, WFR	5 days WFR; 1 mo FFQ	5 V; F(NR)
Sidahmed et al., 2014	*n* = 120 (88% Caucasian, 72% female, mean age 53 years)	24-h recall, FR	6 mos	6 F; 8 V
Tichenor et al., 2015	*n* = 275,864 adults	BRFSS	NR	2 F; 4 V
Torheim et al., 2004	*n* = 502 women age 15–45 in Western Mali	FFQ	7 days	F(NR); V(NR)
Vandevijvere et al., 2010.	*n* = 3245 representative of Belgian population ≥ 15 years old	24-h recall	2 days	F(NR); V (NR)
Vossenaar et al., 2010	*n* = 355 children 8–10 years old; Quetzaltenango, Guatemala	24-h recall	1 day	69 FV
Wolfe et al., 2001	*n* = 31 (included white, African American, and Hispanic persons)	Variety instrument	1 mo	20 F; 24 V
Ye et al., 2013	*n* = 1412 Puerto Rican adults 45–75 years old	FFQ	1 year	27 F; 26 V

Notes: F, fruit; V, vegetable; FV, fruit and vegetable; NR, not reported; ARFS, Australian Recommended Food Score; FFQ, Food Frequency Questionnaire; Q, questionnaire; KOHBRA, Korean Hereditary Breast Cancer; EPIC, European Prospective Investigation into Cancer; WFR, weighted food record; FR, food record; mo, month, mos, months; wk, week; wks, weeks.

**Table 3:** Studies, from the literature, reported as cross-sectional design studies assessing fruit and vegetable (FV) variety.

Citation	Methods: Sample, Measurement Instrument; Timeframe/Frequency	Number of Items or Subgroups	Findings
Almeida-de-Souza et al., 2018	*n* = 412 adolescents, mean age 14.4 years, 52% girls; Portugal Measurement: semiquantitative FFQ Timeframe: ≥once/month, past year	12 F items 15 V items	No inflammation marker differences by F variety; highest tertile of V variety had overall low-grade inflammation; independent of quantity
Azadbakht et al., 2013	*n* = 411 Isfahanian women 12–28 years Measurement: semiquantitative FFQ Timeframe: previous year	2 F subgroups 7 V subgroups	Women consuming breakfast had higher F, V dietary diversity scores
Azadbakht et al., 2012	*n* = 289 Isfahanian women 12–28 years Measurement: semiquantitative FFQ Timeframe: previous year	2 F subgroups 7 V subgroups	Top tertile of energy density had lowest F, V diversity scores; top tertile of DDS had highest V, F diversity scores
Azadbakht et al., 2005	*n* = 295 males 18 and older, 20% were 51 or older; Tehran, Iran Measurement: two 24 h recalls Timeframe: 2 days	2 F subgroups 7 V subgroups	F variety correlated with vitamin C, associated with probability of vitamin A, vitamin C, potassium adequacy; V variety correlated with vitamin A, potassium, vitamin C adequacy
Azupogo et al., 2018	*n* = 187 women age 15–49; rural Ghana Measurement: semiquantitative FFQ Timeframe: past month	27 V items	Increasing trend across VVS tertiles for HRQoL, physical, mental health, physical functioning; significant trend between mental health domain, VVS; higher mental health scores in highest VVS tertile
Bonaccio et al., 2018	*n* = 10,812 adults ≥35 years; Southern Italy Measure: EPIC FFQ Timeframe: once/2 weeks, past year	37 FV items	FV variety positively associated with psychological resilience
Brunt et al., 2008	*n* = 557 Canadian undergraduate students 18–56 years, 60% female; 75% 21 years old or younger Measure: 42 item DVQ Timeframe: past 3 days	5 F items 5 V items	F variety was most limited food group (33% reported ≤1 daily servings); no significant V variety findings
Brunt et al., 2008	*n* = 585 college students 18–56 years Measure: 42-item DVQ Timeframe: past 3 days	5 F items 5 V items 10 FV items	Students living on-campus consumed greater variety of F, V, and FV combined
Burrows et al., 2016	*n* = 25 competitive male rugby players 14–18 years old Measure: ARFS Timeframe: ≥once/week	F NR V NR	Authors state that results indicate need to increase variety within F and V groups
Conklin et al., 2015	*n* = 9580 adults over 50 years old in EPIC-Norfolk (England) Measure: semiquantitative FFQ Timeframe: past year	11 F items 26 V items	Low social class, low education associated with low F, V variety; difficulty paying bills associated with lower F variety in women; combination of low economic resources, being non-married showed greater magnitude of association with F, V variety than social class, education, or paying bills; among women, low social class, difficulty paying bills; being non-married showed double association with lower V variety than for social class and difficulty paying bills
Conrad et al., 2018	*n* = 38,981 adults < 20 years old Measure: 24-h recall Timeframe: 24-h period	V NR	Inverse relationship of V variety with prevalent CHD; living with domestic partner associated with greater V variety, current smoking associated with lower V variety; V variety, amount positively associated; adults consuming dark leafy greens had lower odds of CVD, CHD
de Deus Mendonça et al., 2019	*n* = 3414 adults, older adults; Brazil Measure: Questionnaire (QBrief-F&V) Timeframe: previous 6 months	14 F items 22 V items FV NR	Average of only 2 types FV consumed per day, daily average of 5 servings; authors indicate greater commercial F variety would increase consumption diversity
Giskes et al., 2002	*n* = 654 13–17 years old; *n* = 7695 18–64 year olds; Australia Measure: 24-h dietary recall Timeframe: 24-h period	F NR V NR	The relationship between income and FV variety only significant among adults. Lower-income adults consumed less FV variety than higher-income.
Henry et al., 2006	*n* = 420 low-income, African American mothers aged 18–45 with children < 12 years; St. Paul/Minneapolis, MN; US Measure: FFQ Timeframe: past 4 weeks	20 F items 23 V items	FV variety consumed was higher for women in later stages of change and with higher FV intake
McCrory et al., 1999	*n* = 71 healthy adults 20–80 years old; New England, US Measure: FFQ Timeframe: past 6 months	10 F items 14 V items	V variety was negatively associated with body fatness
Mirmiran et al., 2006	*n* = 286 Tehranian women 18–80 years old Measure: 2 24-h dietary recalls Timeframe: 2 days	2 F subgroups 7 V subgroups	F diversity correlated with vitamin C; F diversity associated with probability of vitamin A, vitamin C, potassium adequacy; V diversity correlated with vitamin A, potassium, vitamin C adequacy
Nour et al., 2017	*n* = 2397 ages 18–34 years old; Australia Measures: 24-h dietary recall Timeframe: 24-h period	6 F subgroups 6 V subgroups	No differences in F variety (consuming ≥2 categories) by age or gender; 18–24 year olds had the lowest V variety, no gender differences; less than ¼ of surveyed reported 3–4 different V
Ramsay et al., 2017	*n* = 2595 children 2–5 years old; 48% male; 55% non-Hispanic white; US. Measure: 24-h dietary recalls Timeframe: 24-h period	3 F subgroups 4 V subgroups	Higher F, V variety scores associated with better dietary quality scores for total F, total V, empty calories subscales; greater differences among those consuming ≥5 different FV.
Robinson et al., 2015	*n* = 66 families with children 8–12 years; New South Wales, Australia Measure: 70-item ARFS Timeframe: NR for ARFS	F NR V NR	F variety intake was most strongly correlated in both parent-child dyads
Tichenor et al., 2015	*n* = 275,864 adults Measure: BRFSS FV questions Timeframe: ≥once/week, previous year	2 F items 4 V items	Less than half of adults consumed F, all V subgroups ≤ once/week. Likelihood of meeting FV variety varied by race/ethnicity, region (*p* < 0.05).
Torheim et al., 2004	*n* = 502 women 15–45 years old; Western Mali Measure: QFFQ Timeframe: 7 days	F NR V NR	High correlation between MAR and food group variety score for V
Vossenaar et al., 2010	*n* = 355 children 8–10 years old; Guatemala Measure: 24-h dietary recall Timeframe: 24-h period	69 FV items	Study sample was not meeting FV variety recommendations
Ye et al., 2013	*n* = 1412 Puerto Rican adults 45–75 years old Measure: semiquantitative FFQ Timeframe: ≥once/month, past year	27 F items 26 V items	Greater FV variety (but not total quantity) associated with higher global cognitive function, executive function, memory, attention scores

Notes: F, fruit; V, vegetable; FV, fruit and vegetable; NR, not reported; VVS, V variety scores, BRFSS, Behavioral Risk Factor Surveillance System; ARFS, Australian Recommended Food Score; FFQ, Food Frequency Questionnaire; QFFQ, Quantitative FFQ; CHD, coronary heart disease; CVD, cardiovascular disease; MAR, mean adequacy ratio (indicator of nutrient adequacy).

**Table 4:** Cohort studies, from the literature, assessing fruit and vegetable (FV) variety.

Citation	Methods	Number of FV Items/Subgroups, Specifics where Possible	Findings
Bhupathiraju et al., 2013	Design: prospective cohort study	11 F items: NR 19 V items: NR	Higher FV intake associated with healthy baseline lifestyle characteristics: higher FV variety scores; higher quantity-adjusted variety scores associated with less smoking, more physically active, higher FV intake. No significant associations found among quantity adjusted FV variety scores and CHD risk.
	*n* = 71,141 women and 42,135 men		
	Measure: Semiquantitative FFQ		
	Timeframe: ≥once per week, average daily intake calculated		
Buchner et al., 2010	Design: ongoing multicenter prospective cohort study	14 F items: NR 8 V subgroups: leafy V; fruiting V; root V; cabbages; mushrooms; grain and pod V; onion and garlic; stalk V 40 FV items: NR 26 V products: NR	V variety inversely associated with lung cancer risk among current smokers; increasing F or V associated with reduced risk of squamous cell carcinomas; independent of quantity, FV variety may decrease lung cancer risk
	*n* = 452187 adult participants; 10 European countries		
	Measure varied by country		
	Timeframe: ≥once per 2 weeks over past 12 months		
Buchner et al., 2011	Design: ongoing multicenter prospective cohort study	14 F items: NR 8 V subgroups: NR 40 FV items: NR 26 V products: NR	No clear association between FV variety consumption and bladder cancer risk; Highest tertile of DDS of FV consumption had marginally significant hazard ratio as compared with lowest (HR = 1.30, 95% CI: 1.00–1.69, *p*-trend = 0.05); individuals consuming higher FV variety were more often women, higher educated, more likely to consume alcohol, more often never smokers, had lower BMIs, and had higher FV consumption
	*n* = 452,185 adult participants; 10 European countries		
	Measure varied by country		
	Timeframe: ≥once per 2 weeks over past 12 months		
Chou et al., 2019	Design: prospective cohort	F: NR V: NR 5 V subgroups: spinach and broccoli; other dark-green V; red and orange V; starchy V; other V	Quantity-adjusted V variety not significantly associated with risk of cognitive decline. However, high diet quality was associated with lower risk of global cognitive decline among elders with high V variety.
	*n* = 436 elders in Taipei		
	Measure: Semiquantitative FFQ		
	Timeframe: intake over previous year		
Cooper et al., 2012	Design: prospective case-cohort	58 F items: NR 59 V items: NR 117 FV items: sum of FV; NR	Greater F variety (0.70 (0.53–0.91)), greater V variety (0.77 (0.61–0.98)), combined FV (0.61 (0.48–0.78)) associated with lower hazard of type 2 diabetes
	*n* = 3704; 653 diabetes cases nested within EPIC and Nutrition-Norfolk; England; age NR		
	Measure: 7-day prospective food diaries		
	Timeframe: 7 days		
Estaquio et al., 2008	Design: part of larger 8-year prospective study	9 F subgroups: apple, pear, other pome F; citrus F; grapes; berries; stone fruits; melon; banana; other tropical F; F juices 10 V subgroups: green salads; leafy V; fruits used as V; root V; green beans, peas; bulb, stem V; flowering V; mushrooms; sprouts; V juices	V variety, education significantly positively related in both men and women; F variety positively associated with education, occupation in men; FV variety scores similar in both sexes; F variety associated with more healthful lifestyle including nonsmoking in men and women, regular physical activity and low alcohol consumption in men; V variety inversely associated with smoking in men
	*n* = 4282 French men and women aged 45–62 years		
	Measure: repeated 24-h dietary recalls over 2 years; used telephone/software assistance system		
	Timeframe: multiple 24-h periods averaged		
Jansen et al., 2004	Design: prospective cohort	7 F types: strawberries; berries; grapes; peaches; cherries; prunes; apricots 27 V types: NR	After excluding first 2 years of followup, F variety associated with reduced cancer risk; V variety but not quantity, inversely associated with total cancer and non-lung epithelial cancer
	*n* = 730 Dutch men aged 65–84 years for 10 years		
	Measure: FFQ		
	Timeframe: Past month		
Ko et al., 2013	Design: cohort	12 F items: NR 25 V items: NR	Dose-response trend for association between low risk of breast cancer and high intake of V; (*p* trend = 0.036); authors posit that inability to separate out cruciferous V from V variety may have diluted impact of V variety
	*n* = 2271 subjects (KOHBRA Study); mean age at study entry 42.5 ± 11.5 years (BRCA carriers), 41.9 ± 10.2 (non-BRCA carriers)		
	Measure: FFQ		
	Timeframe: ≥once per week in year before study		
Leenders et al., 2015	Design: ongoing multicenter prospective cohort study	FV DDS: 49 items (NR) F DDS: 16 items including berries; citrus F; grapes; hard F; stone F V DDS: 33 items including cabbages; fruiting V; grain and pod V; leafy V; mushrooms; onion and garlic; root V; stalk V V subtype DDS: 8 subtypes	Higher FV variety associated with higher absolute consumption of FV. Higher self-reported FV consumption associated with lower risk of colon cancer (HR Q4 vs Q1 0.87, 95%CI 0.75-2.02, *p* for trend 0.02). No association found between FV variety and risk of developing colon cancer. Increased risk of rectal cancer with higher F variety.
	*n* = 521,488 adult participants; 10 European countries		
	Measure varied by country		
	Timeframe: ≥once per 2 weeks over past 12 months		
Oude Griep et al., 2012	Design: prospective population-based cohort study	9 F items: NR 13 V items: NR 22 FV items: NR	F, V variety not related to incident CHD or stroke. Participants consuming greater FV variety were more often women, higher levels of education, less likely to smoke, more likely to be physically active. Strong correlations between variety and total FV intake (Spearman’s r = −0.81, *p* < 0.0001) and F intake (Spearman’s r = 0.72, *p* < 0.001). Positive association of variety with vitamin C, carotenoids, flavonoids, and dietary fiber intake.
	*n* = 20069 (8988 men, 11,081 women); Dutch		
	Measure: FFQ		
	Timeframe: ≥once per two weeks in previous year		

Notes: F, fruit, V, vegetable; FV, fruit and vegetable; NR, not reported; CHD, cardiovascular heart disease, DDS, dietary diversity score; KOHBRA, Korean Hereditary Breast Cancer (KOHBRA) Study.

**Table 5:** Case-only and case-control studies assessing fruit and vegetable variety in the literature.

Citation	Methods	Number of FV Items/Subgroups	Findings
Ghadirian et al., 2009	Design: case-only, breast cancer	VF, number, specific items NR	Strong significant interaction between BRCA mutations and VF diversity between upper and lower quartiles
	*n* = 739 women in original cohort; mean age 50.5 ± 10.2 years for BRCA carriers, 53.4 ± 7.7 for non-BRCA carriers		
	Measure: interviewer administered FFQ		
	Timeframe: ≥once per week in year prior to diagnosis or enrollment for matched controls		
McCann et al., 1994	Design: case-control	38 F items; specific items NR	Female cases had slightly higher (non-significant) F diversity than controls; for both men and women, F diversity was positively associated w V diversity; among women, F diversity strongly related to meat diversity—trends in risk associated w F diversity among women not statistically significant, all models suggested F diversity to be risk elevating rather than protective; female cases had lower V diversity than controls (*p* < 0.05)
	*n* = 428 adults, (205 men, 223 women), colon cancer cases; all Caucasian; 3 counties in Western New York		
	Measure: 2.5 h in-person interview including FF instrument	20 V items; specific items NR	
	Timeframe: 12 months preceding diagnosis, or preceding interview for controls		
Randall et al., 1989	Design: case-control	F; number, specific items NR	Total, F, and V diversity scores associated with fiber, vitamin A, and vitamin C intake.
	*n* = 428 adults, (205 men, 223 women), colon cancer control subjects; Western New York	V, number, specific items NR	
	Measure: 2.5 h in-person interview including FF instrument		
	Timeframe: >once per month over past 12 months		

Notes: F, Fruit; V, Vegetable; FV, fruit and vegetable; NR, not reported; BRCA, BReast Cancer gene; FFQ, Food Frequency Questionnaire; FF, food frequency; VF, vegetable and fruit.

## Appendix A. Protocol

1. Title: Protocol of A Scoping Review of the Operationalization of Fruit and Vegetable Variety
2. Registration N/A
3. Contact information
  Allison N. Marshall
  Corresponding Author: Allison Marshall, Allison.n.marshall.5@gmail.com
  University of Texas Health Science Center Houston School of Public Health Austin, Michael and Susan Dell Center for Healthy Living, 1616 Guadalupe St. Ste 6.300, Austin, Texas 78745
  Alexandra van den Berg ^1^
  Nalini Ranjit ^1^
  Deanna M. Hoelscher ^1^
  Contributions.
  Guarantor: A.N.M.
  All authors contributed to development of selection criteria, data extraction criteria. AM and D.M.H. developed search strategy. All authors contributed expertise on fruit and vegetable consumption and measurement. A.N.M. drafted protocol. All authors read, provided feedback, and approved the final protocol.
4. Amendments
  If we need to amend this protocol, dates, amendments, and rationale will be presented in this section. Changes will not be incorporated into the protocol.
5. Funding Support
  This scoping review was partially funded by the Michael and Susan Dell Foundation through the Michael and Susan Dell Center for Healthy Living. It was completed as part of dissertation work to satisfy degree requirements for the PhD program.
6. Rationale
  Intake of fruit and vegetables (FV) is recognized as crucial for optimal health in childhood and adulthood and is critical for proper physical and psychosocial development and functioning for children and adolescents. The World Health Organization (WHO) recognizes inadequate FV consumption as a factor for 14% of gastrointestinal cancer deaths, 11% of ischemic heart disease deaths, and 9% of stroke deaths worldwide, and considers low FV intake as one of the top 10 risks for death worldwide. FV intake is especially critical during childhood and adolescence both because of rapid growth, and because lifestyle habits from childhood tend to track into adulthood. Additionally, low FV intake is associated with higher BMI and higher risk of obesity in childhood, and childhood obesity is linked to excess weight in adolescence and adulthood. Most people are not consuming enough fruit and vegetables, and few studies measure variety.
  Fruit and vegetable consumption recommendations generally focus on amount; in addition to amounts, the U.S. Dietary Guidelines for Americans 2015–2020 also include a recommendation for fruit and vegetable variety over the course of a week. Although few studies assess variety, there is variation across existing studies. There is a gap in the literature regarding fruit and vegetable variety.
7. Objectives
  The objectives of our study are to systematically review the literature to identify currently available evidence operationalizing fruit and vegetable variety to summarize, compare, and critically evaluate the operationalization of variety.
8. Eligibility Criteria
  Studies will be selected according to the criteria outlined below:
  **Study designs** All study designs are eligible for inclusion in this systematic review. Etiologic, intervention, and determinant studies are eligible to be included;
  **Participants** We will include studies examining human population ages 2 years old and older;
  **Interventions** Any type of intervention is eligible for inclusion in this review;
  **Setting** There will be no restrictions by type of setting;
  **Language** We will include articles reported in the English language.
9. Information Sources
  We will search PubMed, Medline, PsycINFO all available years. Searches for headings and key words will be conducted using combinations of the following terms: Fruit OR fruits OR vegetable OR vegetables OR diet OR dietary OR nutrition AND variety OR diversity AND measure OR measurement OR assess OR assessment. To ensure a comprehensive review, reference lists of included papers will be scanned for additional records. In addition, papers from the authors’ existing literature base will be included. A list of included items will be circulated to the systematic review team.
10. Search Strategy
  Etiologic, determinant, and intervention studies will be sought. No study design or date limits will be imposed on the search. Only publications published in English will be included. All available years of PubMed, Medline, PsycINFO will be searched. The specific search strategies will be created based on guidance from a health sciences librarian with expertise in systematic reviews.
  Draft of PsycInfo search—Ovid interface
  1. fruits.mp. [mp=title, abstract, heading word, table of contents, key concepts, original title, tests & measures, mesh]
  2. fruit.mp. [mp=title, abstract, heading word, table of contents, key concepts, original title, tests & measures, mesh]
  3. vegetable.mp. [mp=title, abstract, heading word, table of contents, key concepts, original title, tests & measures, mesh]
  4. vegetables.mp. [mp=title, abstract, heading word, table of contents, key concepts, original title, tests & measures, mesh]
  5. 1 or 2 or 3 or 4
  6. diet.mp. [mp=title, abstract, heading word, table of contents, key concepts, original title, tests & measures, mesh]
  7. dietary.mp. [mp=title, abstract, heading word, table of contents, key concepts, original title, tests & measures, mesh]
  8. nutrition.mp. [mp=title, abstract, heading word, table of contents, key concepts, original title, tests & measures, mesh]
  9. 6 or 7 or 8
  10. diversity.mp. [mp=title, abstract, heading word, table of contents, key concepts, original title, tests & measures, mesh]
  11. variety.mp. [mp=title, abstract, heading word, table of contents, key concepts, original title, tests & measures, mesh]
  12. 10 or 11
  13. measure.mp. [mp=title, abstract, heading word, table of contents, key concepts, original title, tests & measures, mesh]
  14. assess.mp. [mp=title, abstract, heading word, table of contents, key concepts, original title, tests & measures, mesh]
  15. 13 or 14
  16. 5 and 9 and 12 and 15
  17. limit 16 to ((160 preschool age <age 2 to 5 yrs> or 180 school age <age 6 to 12 yrs> or 200 adolescence <age 13 to 17 yrs> or "300 adulthood <age 18 yrs and older>" or 320 young adulthood <age 18 to 29 yrs> or 340 thirties <age 30 to 39 yrs> or 360 middle age <age 40 to 64 yrs> or "380 aged <age 65 yrs and older>" or "390 very old <age 85 yrs and older>") and english and human)

11a Data Management
  Records for all articles resulting from the search strategy applied in each of the three databases will be exported to Refworks. Duplicates will be removed, and titles and abstracts scanned for relevancy to identify a list of potentially relevant studies. Articles not meeting the inclusion criteria will be removed and reasons for exclusion noted. Titles and abstracts potentially meeting the inclusion criteria will be screened in full.
11b Selection Process
  The lead review author will independently screen titles and abstracts. We will seek additional information from study authors and from review co-authors as needed to resolve questions about eligibility. We will record the reasons for excluding studies. Review authors will not be blind to journal titles or study authors or institutions.
11c Data Collection Process
  Using a standardized abstraction form, demographic information of participants, methodology including study design and measurement instruments, purpose of study, and reported outcomes will be abstracted. We will contact study authors to resolve any uncertainty and review co-authors will be consulted to resolve uncertainty.
12 Data Items
  We will extract the study design, sample size, participant demographics including geographic location, type and length of measurement instrument, number of fruit and vegetable items and details of specific classification where available, structure of basis for fruit and vegetable classification, length of time assessed, minimum amount of consumption required to be considered for variety, major findings of the studies. Any missing data pertaining to eligibility for inclusion will be noted and the record will proceed to full-text review; any missing data will be reported in the table as “not reported.” Because the purpose of this systematic review is to compare differing operationalizations of fruit and vegetable variety, the degree of detail which is reported is relevant to report and compare.
13 Outcomes and Prioritization
  Primary outcomes include the number of fruit and vegetable items, classification of items, basis of classification, study methodology, and purpose of studies.
  Secondary outcomes include correlates of variety consumption and health outcomes identified.
14 Risk of Bias
  Because the purpose of this systematic review is to compare operationalization of fruit and vegetable variety, risk of bias in studies is not the focus of this review. Risk of bias of the review due to restriction to English language and variations in classification of fruit and vegetables by country are to be listed as limitations.
15 Analysis
  Descriptive comparisons will be presented, further meta-analysis will not be conducted. Findings of the systematic narrative synthesis will be presented in text and tabular format to summarize characteristics, findings of included studies.
